# Alkaloids of narrow-leaved lupine as a factor determining alternative ways of the crop’s utilization and breeding

**DOI:** 10.18699/VJ20.656

**Published:** 2020-10

**Authors:** M.A. Vishnyakova, A.V. Kushnareva, T.V. Shelenga, G.P. Egorova

**Affiliations:** Federal Research Center the N.I. Vavilov All-Russian Institute of Plant Genetic Resources (VIR), St. Petersburg, Russia; Federal Research Center the N.I. Vavilov All-Russian Institute of Plant Genetic Resources (VIR), St. Petersburg, Russia; Federal Research Center the N.I. Vavilov All-Russian Institute of Plant Genetic Resources (VIR), St. Petersburg, Russia; Federal Research Center the N.I. Vavilov All-Russian Institute of Plant Genetic Resources (VIR), St. Petersburg, Russia

**Keywords:** narrow-leaved lupine, alkaloids, domestication, breeding, feed, food, green manure, varieties, pharmacology, genetic and genomic resources, люпин узколистный, алкалоиды, доместикация, селекция, кормовые, продовольственные, сидеральные сорта, фармакология, генетические и геномные ресурсы

## Abstract

Narrow-leaved lupine (Lupinus angustifolius L.), a valuable leguminous crop adapted to a wide range of climatic conditions, has a very short history of domestication. For many centuries it was used mainly as a green manure, since the success and prospects of the multi-purpose use of the species depend on its breeding improvement, in particular, on a particular concentration of alkaloids in seeds and green mass. The first varieties of scientific breeding were created only in the 1930s after the appearance of low-alkaloid mutants. Despite wide prospects for use in various areas of the national economy, unstable productivity and susceptibility to diseases hinder the production of this crop. Obviously, breeders deal only with a small part of the gene pool of the species and limited genetic resources, using mainly low-alkaloid (sweet) genotypes to create new varieties. The genetic potential of the species can be used more efficiently. At the same time, it is rational to create highly alkaloid (bitter) varieties for green manure, while food and feed varieties should not lose their adaptive potential, in particular, resistance to pathogens, due to the elimination of alkaloids. In this regard, it seems to be a productive idea to create ‘bitter/sweet’ varieties combining a high content of alkaloids in the vegetative organs and low in seeds, which can be achieved by regulating the synthesis/transport of alkaloids in the plant. The paper discusses the current state of use of the species as a green manure, fodder, food plant. Information is given on the quantity and qualitative composition of narrow-leaved lupine alkaloids, their applied value, in particular, fungicidal, antibacterial, insecticidal, the use of lupine alkaloids as active principles of drugs. Along with promising breeding considerations, the possibility of using technologies for processing raw high-alkaloid materials with the accompanying extraction of valuable ingredients for pharmaceuticals is discussed. Information is briefly presented about the genomic resources of the species and the prospects for their use in marker-assistant selection and genome editing.

## Abstract (ru)

Люпин узколистный (Lupinus angustifolius L.) – ценная зернобобовая культура, адаптирован- ная к широкому спектру климатических условий и имеющая непродолжительную историю доместикации. В течение многих веков его употребляли преимущественно как сидеральное растение, поскольку успех и перспективы многоцелевого использования вида зависят от его селекционного улучшения, в частности от содержания определенного уровня алкалоидов в семенах и зеленой массе. Первые сорта научной селекции были созданы в 1930- х гг., после выявления низкоалкалоидных мутантов. Производство этой культуры сдер- живается нестабильной урожайностью и подверженностью болезням. Очевидно, что селекционеры имеют дело лишь с небольшой частью генофонда вида и ограниченными генетическими ресурсами, используя для получения новых сортов преимущественно низкоалкалоидные (сладкие) генотипы. Генетический потенциал вида можно задействовать эффективнее. При этом сидеральные сорта рационально создавать высокоалка- лоидными (горькими), а продовольственные и кормовые за счет элиминации алкалоидов не должны терять адаптивные свойства, в том числе устойчивость к патогенам. В этом отношении продуктивной идеей пред- ставляется выведение сладко-горьких сортов, сочетающих высокое содержание алкалоидов в вегетативной массе и низкое – в семенах, чего можно добиться путем регулирования синтеза/транспорта алкалоидов в растении. В обзоре рассмотрены современное состояние использования вида в качестве сидерального, кормового, пищевого растения. Приведены сведения о количестве и качественном составе алкалоидов лю- пина узколистного, их прикладном значении, в частности фунгицидной, антибактериальной, инсектицид- ной функциях, применении отдельных алкалоидов люпина в качестве действующих начал лекарственных средств. Наряду с селекционным улучшением культуры обсуждаются возможные технологии переработки высокоалкалоидного сырья с сопутствующим извлечением ценных ингредиентов для фармацевтики. Кратко представлены сведения о геномных ресурсах вида и перспективах их использования в маркер-опосредо- ванной селекции и при редактировании генома.

## Introduction

The narrow-leaved lupine (Lupinus angustifolius L.), also known as the blue lupine, is one of the three Lupinus spp. cultivated in Russia. Along with white (L. albus L.) and yellow (L. luteus L.) lupines, it is a valuable pulse crop, whose seeds contain 30–40 % of protein, up to 40 % of carbohydrates, 6 % of oil, numerous minerals, vitamins, and other beneficial ingredients, ranking this species among the most important crops of the present and the future.

Today, L. angustifolius is a leader among other cultivated lupine species in the cropping area occupied worldwide. It is widely cultivated in Northern Europe, countries of the ex-USSR, the United States, and New Zealand. The world’s leading producer and exporter of this crop is Australia, where the areas under narrow-leaved lupine reach 0.6–0.7 million hectares, and large funds are invested in its research and breeding. In Russia, in 2018, its production area was 35,000 ha, which is not much considering the size of the country. However, the Russian Federation is still among the top ten producers of this crop (http://www.fao.org/faostat/en/#data/QC).

Lupinus angustifolius is the most early-ripening and most plastic crop species among those produced in Russia and the only one adapted to high northern latitudes – up to 60° N. It grows on acidic sandy soils deficient in nitrogen and phosphorus, and is a powerful nitrogen accumulator. Its growing season lasts from 70 to 120 days, depending on the cultivar and the climate. Total active temperatures of 1900 °С and precipitation amount of 200–250 mm from germination to maturity are enough for successful seed production. The crop endures a decrease in air temperature down to –9 °C (Kuptsov, Takunov, 2006).

Potential uses of narrow-leaved lupine have not yet been practiced to the fullest extent. Historically, this crop was grown for green manure and animal feed. These days, its nutritional, pharmacological and phytoremedial properties are coming into the sphere of interest, as well as its use as a feed in aquaculture. The prospects of its utilization as a source of bioethanol (Kuznetsova et al., 2015) and natural fiber (Kozlowski, Manys, 1997) are discussed.

The uses of narrow-leaved lupine depend on the presence of secondary metabolites in its seeds and biomass, especially quinolizidine alkaloids responsible for bitter taste and toxic to both humans and animals. Polymorphism of the species’ gene pool in the content of such compounds makes it possible to develop cultivars for a specific purpose. High-alkaloid genotypes are promising as green manure plants and producers of alkaloids for pharmaceutics and medicine, while low-alkaloid ones may be used for food and feed purposes.

This review attempts to analyze different applications of narrow-leaved lupine genetic resources depending on the content of alkaloids in the genotypes, describe lupine alkaloids and their practical worth, assess the need for targeted breeding of specialized cultivars, survey genetic and genomic resources promising for breeding, and discuss possible technologies capable of expanding the crop’s economic potential.

## Domestication and breeding history

The center of origin for narrow-leaved lupine is the Mediterranean region. L. angustifolius occurs as a wild plant much more frequently than other lupine species in the Old World and is still widespread across the entire Mediterranean basin. It has also naturalized in South Africa and South-Western Australia (Gladstones et al., 1998). The species dispersed from the Mediterranean center to Central European countries, winning special recognition in Germany and Poland. In Russia, narrow-leaved lupine became known only in the early 20th century.

For thousands of years lupine has been used for green manure and animal feed. Before feeding the animals, lupine seeds were soaked in water, with several changes, in order to remove alkaloids.

The revolution in lupine breeding was observed in 1926– 1928, when Reinhold von Sengbusch, a German botanist, discovered natural low-alkaloid mutants. It helped to reduce the alkaloid content in the seeds of L. albus, L. luteus and L. angustifolius from the traditional 1–3 % to 0.02 % and less (Sengbusch, 1931). Since that time, the breeding of low-alkaloid (sweet) lupine cultivars for animal feed has been gaining progress. Initially, such cultivars emerged in Germany, then in Sweden, Denmark and Poland. In the USSR, the first natural low-alkaloid mutants were developed by scientists at VIR and plant breeders of the Novozybkov and Minsk Experiment Stations (Anokhina et al., 2012). In Australia, where lupines were introduced in the 1960s to improve crop rotations and reclaim sandy soils, the first sweet cultivar adapted to the local environments was released soon afterwards, in 1967, and large-scale lupine grain production started in 1973–1974 (Gladstones, 1982).

**Fig. 1.**
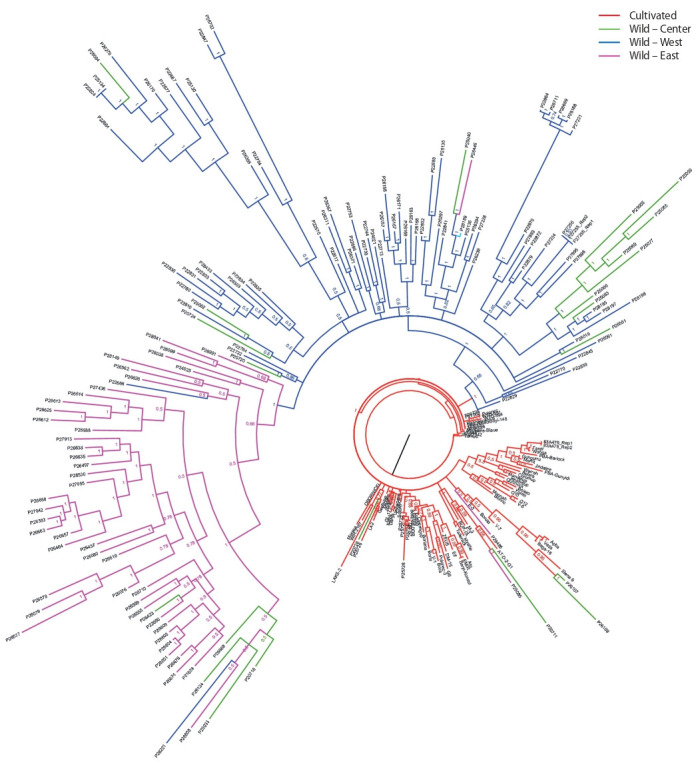
The phylogenetic tree for wild and cultivated narrow-leaved lupine accessions produced on 11,690 SNPs using MrBayes v3.2.2, according to (Mousavi Derazmahalleh et al., 2018). Wild accessions from the central part of the Mediterranean region (21 accessions) are presented in green color; from western Mediterranean (77 accessions) in navy blue; from eastern Mediterranean (49 accessions) in pink; and cultivated forms (87 accessions) in red.

Same as with most of the crops, the genetic diversity of domesticated narrow-leaved lupine forms is smaller than that of wild populations and landraces, and plant breeders have employed only a minor part of this diversity (Berger et al., 2012a, b). Whole genome sequencing of 146 wild and 87 cultivated accessions from different genebanks over the world ascertained that the genomic diversity in modern cultivars is thrice smaller than in wild populations (Mousavi-Derazmahalleh et al., 2018) (Fig. 1). It should be mentioned that 90 years that have passed since the development of the first low-alkaloid cultivars is quite a short time for an agricultural crop, and the process of introducing this species into cultivation cannot be regarded as finalized.

Russian breeding centers working with narrow-leaved lupine are the All-Russian Research Institute of Lupine, Nemchinovka Federal Research Center, Belogorka Research Institute of Agriculture, Moscow Timiryazev Agricultural Academy, etc. Presently, there are 27 cultivars of narrow-leaved lupine listed in the State Register for Selection Achievements Admitted for Usage (http://reestr.gossortrf.ru/reestr.html).

Cultivars released in the early stages of the lupine breeding history were, as a rule, high-alkaloid, but those developed later, due to the selection of genotypes with reduced alkaloid content, were predominantly low-alkaloid (Anokhina et al., 2012). According to the standards accepted by a number of European countries and Australia, the content of alkaloids in seeds intended to be used for food or feed purposes (sweet) must not exceed 0.02 % of their dry weight (Frick et al., 2017). In Russia, fodder lupine seeds should have the percentage of alkaloids from 0.1 up to 0.3 % of the seed dry weight (GOST R 54632-2011, 2013), and in those for human food their content is restricted to 0.04 %, in line with the existing technical specifications developed at the All-Russian Research Institute of Lupine (TU-9716-004-0068502-2008).

## Lupine as a green manure crop

With the green biomass yield of 45–60 t/ha, lupine is able to accumulate 100–300 kg/ha of ecologically safe biological nitrogen in its biomass, which is comparable with animal manure. Thus, the conditions are created for a stable or increased supply of the soil with organic matter, so that its physical and chemical properties improve, and the phytosanitary state of subsequent plantings is upgraded (Kuptsov, Takunov, 2006).

Due to its deeply penetrating roots and high dissolving capacity of root excretions, lupine assimilates phosphorus, potassium, calcium, magnesium and other elements, contributing to their intensified circulation in the topsoil and subtopsoil horizons. On average, one hectare of lupine plants leaves to the next crop, in addition to nitrogen, 30 kg of phosphorus and 50 kg of potassium (Yagovenko et al., 2003).

The demand for narrow-leaved lupine as a green manure plant continues to grow. The yield of winter rye sown into gray forest soil on a green manure fallow after lupine, without fertilizers, increases by 0.5–1.0 t/ha (Gresta et al., 2017). Besides, the alkaloids contained in the plowed green biomass produce a decontaminating effect on the soil, thus reducing the negative impact inflicted on subsequent crops by their diseases and pests, such as various root rots for cereals or scab, Rhizoctonia rot and golden nematode for potato (Evstratova et al., 2012). This phenomenon is undoubtedly interesting as a protective tool against fungal disease agents, and calls for further research into the mechanism of alkaloid activities (Anokhina et al., 2008; Romeo et al., 2018). Hence, high alkaloid content becomes a preferable trait in green manure cultivars, which serves to simplify breeding schemes.

Today, main requirements to lupine cultivars grown for green manure are high dry matter yield, rapid growth, and increased nitrogen-fixing activity. The latest cultivars developed by Russian breeders – ‘Oligarkh’, ‘Metsenat’ and ‘Akkord’ (Belogorka Research Institute of Agriculture) – contain 1.5 % of alkaloids in seeds and 0.7 % in the dry matter of green biomass, produce high yields of biomass (31–37 t/ha), demonstrate rapid initial growth and prolific foliage, and are ready for plowing into the soil in the second half of July, i. e., 50–60 days after sprout emergence (Lysenko, 2019).

## Fodder qualities of narrow-leaved lupine

Lupine fodder is considered a good alternative to soybean: digestibility and feed energy coefficients of lupine proteins are level with those of soybean and exceed those of pea, while the yield of lupine in the European part of Russia is 1.5–2.0 times higher than soybean yield. Many European countries do not produce soybean, so they are forced to export it, mainly from South America, but the production areas under lupine in Europe have good prospects for expansion. According to the estimates by experts, the cost price of lupine grain production is twice lower than that of soybean grain. Besides, narrow-leaved lupine produces higher yields at lower energy costs than soybean: 840.7– 846.6 MJ/100 kg (Feed Production Handbook…, 2014).

The value of lupine as a fodder crop is all the more palpable in view of the fact that not only grain but also its green biomass, with 18–23 % of crude protein and up to 14 % of sugar in dry matter, is readily consumed by all kinds of farm animals. Lupine is used for feed as freshly cut plants, in crushed grain and compound feeds, as silage and haylage, as a component of cereal and legume haylage mixtures, etc. (Kuptsov, Takunov, 2006). Its green biomass is numbered among highly nutritional succulent feeds, distinguished for its good digestibility and feed consumability. Lupine straw contains up to 7 % of protein, which is the evidence of its higher feeding value than the straw of cereal crops. It may be added to silages made of the biomass of other crops. Lupine regrowth may be used for grazing, especially as far as swine and sheep are concerned. It is much more nourishing than the stubble of cereals; it is even compared with grassy legume pastures. The practice of pasturing sheep and calves on harvested fields where grain and fodder lupine cultivars were grown is widespread in Australia (Gladstones, 1970). It should be mentioned that the regrown lupine stubble is not the only valuable grazing resource in a harvested field: leftover seeds are also a bonus – their losses at harvesting range from 150 to 400 kg/ha (Truter et al., 2015).

The lupine grain contains high enough amounts of tocopherol (3.9–16.2 mg%) and carotenoids (10–21 mg%), and 90 % of the latter is carotene. This is especially important for aquaculture, as many fish species cannot exist without carotenoids (Korol, Lakhmotkina, 2016a).

## Narrow-leaved lupine as human food

When eight crops were discussed in the context of their eligibility as major sources of plant protein for Western Europe, considering their agronomic advantages, prospects for quick improvement, yield and quality of protein, technological aspects, functional and nutritional properties, lupine and pea were recognized as preferential over potato, triticale, alfalfa, etc. (Linnemann, Dijkstra, 2002; Dijkstra et al., 2003).

Beginning from the late 20th century, lupine seeds have been widely used as ingredients by food industries in a number of European countries, Canada, the U.S., Chile, Australia, and to a much lesser extent in Russia and Belarus. Each year Europe consumes about 500,000 tons of lupine-containing food products, including lupine flour, lupine bran, lupine curd (tofu), etc. used as ingredients of bread, pastry, pasta, dressings, milk substitutes, soybean substitutes in sausages, etc. Traditional for Southern Europe is a popular ‘Lupini’ snack, looking and tasting like popcorn or cornflakes (Yáñez, 1990).

Lupine products are regarded as functional food. They contain little fat and starch. Their glycemic index is low, which is taken into account by nutrition strategies to control obesity, diabetes and cardiovascular diseases. Besides, they are gluten-free, which is important for celiac patients, so they are a valuable reserve to widen the range of foodstuffs for this category of the population (Krasilnikov et al., 2010; Pankina, Borisova, 2015). Lupine proteins possess emulsifying and foaming capacities, which allow them to substitute butter and eggs in cookery (Kohajdorová et al., 2011). Lupine seeds are rich in ferritin, an iron-storing protein (Lucas et al., 2015).

Besides, the grain of lupine owes its functional value in human nutrition to dietary fibers whose content reach 41.5 % (Lakhmotkina, 2011; Lucas et al., 2015). Favorable properties to food products are rendered by lupine oil, with its well-balanced fatty acid composition and an optimal ratio of omega-3 and omega-6 acids – from 1 : 1.7 to 1 : 10.8 (Sedláková et al., 2016).

Phenolic components and flavonoids in narrow-leaved lupine demonstrate antioxidant activity (Martínez-Villaluenga et al., 2009), reduce the risk of cardiovascular diseases through their protective effect on blood vessels (Oomah et al., 2006), and deter the development of some types of cancer, specifically the rectal cancer (Lima et al., 2016). Unlike soybean, lupine contains small amounts of phytoestrogens and less antinutrients, such as phytic acid, oligosaccharides, trypsin inhibitors, lectins, tannins and saponins, than other legumes (Martínez-Villaluenga et al., 2009).

Lupine seeds contain lutein and zeaxanthin – compounds known for their ability to hinder retinal degradation (Fryirs et al., 2008; Wang et al., 2008).

The most popular lupine-based product with food industry is lupine flour. It is rich in lysine but poor in sulfurcontaining amino acids, such as methionine and cysteine, therefore it can serve as a good supplement to lysine-deficient wheat flour (Dervas et al., 1999). Adding 10 % of lupine flour to bread, pasta or bakery products will not only increase their functional value but also improve their texture, flavor and color, concurrently extending their shelf life (Pollard et al., 2002).

In Russia, technologies have been proposed to make pastas and fillings from lupine grain (Pankina, Borisova, 2015). Properties and effects of dietary fibers from lupine hulls are being studied in the context of their use as functional ingredients in some meat products, such as intermediate minced poultry meat (Lakhmotkina, 2011), etc.

Utilization of lupine for food purposes is growing worldwide. In Australia it is called the ‘superfood’ of the 21st century. It is expected that in the nearest future major international markets of nutriceutics (in the European Union, United States and Japan) will rise due to the onset of chronic cardiovascular diseases, nervous disorders, and type 2 diabetes. A potentially huge market demand for lupine-based products also exists among vegetarians, vegans, and people intolerant to gluten, soy, milk or eggs as well as in the growing sector of those who favor healthy diets (Lucas et al., 2015).

## Alkaloids in narrow-leaved lupine

**Composition, variability and toxicity**

Alkaloids are products of secondary metabolism. Unlike primary metabolites, their functional significance is not on the level of a cell but on the level of a whole plant. Most often these compounds perform ‘ecological’ functions, i. e., protect the plant from various pests and pathogens, ensure interactions among plants and between plants and other organisms within an ecosystem, etc. (Borisova et al., 2020).

Different Lupinus species possess a unique alkaloid profile. Usually it consists of 4–5 major alkaloids and several minor ones. The alkaloid composition of a plant is handy for taxonomic purposes (Frick et al., 2017).

Polymorphism of the narrow-leaved lupine gene pool in the content of alkaloids in seeds was uncovered by Polish researchers who studied 329 accessions from the lupine collection: 0.0005–2.8752 % (Kamel et al., 2016). A common feature of all species is a high alkaloid content in seeds (up to 4 %) and a lower content in green biomass (up to 1.5 %). In flowers up to 2.5 % is observed, while in roots their amount is minimal (Lee et al., 2007).

Prevailing alkaloids in narrow-leaved lupine seeds are lupanine (65–75 % of the total alkaloid content), angustifoline (10–15 %) and 13-hydroxylupanine (10–15 %). Minor levels are demonstrated by sparteine and lupinine (Blaschek et al., 2016). These values may vary depending on the genotype and its locality. The concentration of alkaloids in plant organs and their correlation can change under the effect of different growing conditions (Cowling, Tarr, 2004). Even in low-alkaloids cultivars their content is prone to variations within quite an extensive range, exceeding threshold limit values (Romanchuk, Anokhina, 2018). Lupines growing at high latitudes were found to contain less alkaloids than those in southern areas (Gresta et al., 2017).

Accumulation of alkaloids in different plant organs is not simultaneous. In the branching phase, when photosynthesis is especially active, the highest alkaloid content is observed in leaves. In the flowering phase, an intensive efflux of alkaloids occurs from the vegetative organs of a plant to the generative ones, where their content reaches their maximum by the beginning of pod maturation. Each phase of plant development is characterized by its own qualitative composition of secondary metabolites, including alkaloids. Hydroxylupanine dominates at the start of branching, and lupanine at the time of flowering and pod maturation. By the seed ripening period, the alkaloid content in seeds is 5–10 times higher than in green biomass (Akritidou et al., 2015). Sparteine and lupanine are the most toxic, followed in descending order by lupinine, hydroxylupanine and angustifoline (Allen, 1998).

Interestingly enough, when a plant has been mechanically injured, the amount of alkaloids in it grows fourfold. The injury, in this case, mimics the bite of an insect, which may serve as an evidence of the protective functions performed by alkaloids. With this in view, lupanine inflicts the strongest toxic effect on sucking insects (Wink, 1983, 1992).

Metabolomic profiling may prove an effective approach to the assessment of alkaloid biosynthesis activity in the species’ gene pool and the impact of diverse abiotic and biotic environmental factors on this process. Studying metabolomic profiles in wild lupine forms will help to identify or specify the role of individual alkaloids in the species’ adaptation to changing environmental conditions (Romanchuk, Anokhina, 2018).

**Practical importance of narrow-leaved lupine alkaloids**

Since long ago alkaloids have been extensively applied in medicine, pharmacology, veterinary and other sectors. In the first place, they are used as effective agents in pharmaceuticals to provide complex treatment of many dangerous diseases, cancer included (Kruglov et al., 2015; Ding et al., 2019). They prevent the onset of various degenerative pathologies, binding free radicals and metal ions that activate enzymes of oxidative reactions. They also inhibit the growth and development of fungi, protozoa, bacteria, etc. (Ding et al., 2019).

Sparteine has the widest application. It decreases the level of glucose in an organism and initiates insulin secretion (Sgambato et al., 1986), exerts a mild analgesic effect, and acts as an anticonvulsant and antiepileptic (Villalpando- Vargas, Medina-Ceja, 2016). Together with lupanine and hydroxylupanine, it is included in the composition of antiarrhythmic drugs. Such antiarrhythmic effect weakens in the descending order of sparteine–lupanine–hydroxylupanine (Blaschek et al., 2016). Among the Class IA medicaments against tachyarrhythmia there is a combined drug, known as Pulsonorma, which incorporates in its composition ajmaline, sparteine, antazoline and phenobarbital (Ivashev et al., 2013).

Lupanine is a very active neurotransmitter for nAChR (nicotinic acetylcholine receptors) which play a decisive role in neuron signal transmission. The data were obtained on its ability to increase insulin secretion (Wiedemann et al., 2015). It may be used as source material for the synthesis of other alkaloids which are very difficult to produce artificially (Wink, 1987).

**Fig. 2.**
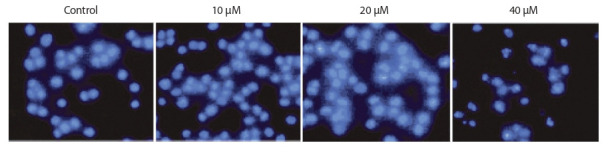
The intensity of angustifoline-induced apoptosis in malignant tumor cell culture from the human large intestine grows with an increase of angustifoline concentration in the medium, according to (Ding et al., 2019). Fluorescent microscopy with DAPI staining.

Angustifoline on the cell culture of a malignant tumor in the human large intestine (line COLO-205) induced autophagy in tumor cells, apoptosis processes and interruption of the cell cycle in the G2/M stage, so it may be regarded as an antineoplastic agent (Ding et al., 2019) (Fig. 2).

Lupinine demonstrates moderate antiglycation activity, without any cytotoxic effect (Abbas et al., 2017). It is also characterized by strong insecticidal activity (Campbell et al., 1933).

Allelopathic effects of lupine alkaloids are confirmed again and again (Wink, 1993), for example, by their antimicrobial activity in the culture of Staphylococcus aureus, Escherichia coli, Pseudomonas aeruginosa Mig., Bacillus subtilis Cohn., Klebsiella pneumoniae Trevis., in concentrations 3–4 orders lower than that of antibiotics (Erdemoglu et al., 2007).

An in vivo trial on lupine-fed goats showed moderate but credible activity of a lupine seed extract against the nematodes Haemonchus contortus and Teladorsagia circumcincta (Dubois et al., 2019).

There is a lot of evidence to the antifungal effect of alkaloids. Fusarium resistance of high-alkaloid lupine cultivars was shown to be higher than that in low-alkaloid ones, and an increased alkaloid content in plant cells was reported in response to the infection by causative agents. Purified lupine alkaloids may be used for pre-sowing treatment of legume crop seeds to raise their resistance to anthracnose, Fusarium, and other fungal diseases. The advantage of alkaloids over synthetic fungicides is their biodegradation and lesser toxicity (Anokhina et al., 2008).

## New breeding trends or old processing techniques?

There are five known genes reducing alkaloid content in narrow-leaved lupine seeds: iuc (iucundus), es (esculentus) (Hackbarth, 1957), dep (depressus) (Hackbarth, Troll, 1956), a1, a2, a3 (angustifolius) (Mikolajczyk, 1966), and tant (tantalus) (Zachow, 1967). The iuc gene determines a reduction in alkaloid concentration approximately to 0.06 % dry weight, dep is responsible for very low content of alkaloids (ca. 0.01 %), while es governs their intermediate concentration (Hackbarth, Troll, 1956). Each stage in the synthesis of alkaloids is controlled by certain alleles, capable of independent mutations and recombinations. Non-allelic mutations are possible: they have a similar phenotypic effect, leading to a low content or absence of alkaloids (Anokhina, 1975). The discovery of complementary gene interactions made it possible to produce the first absolutely alkaloid-free forms by uniting the genes of two non-allelic recessive mutants in one genotype (Sengbusch, 1942). Thus, the absence or low content of alkaloids is a complex quantitative trait of polygenic nature with free complementation between its non-allelic complementary genes (Anokhina, 1975), which is a serious obstacle for the crop’s breeding and seed production.

It was observed in the process of breeding sweet cultivars of narrow-leaved lupine that they were considerably less resistant to diseases and pests than bitter ones: their susceptibility to insect attacks increased as well as, accordingly, vulnerability to virus diseases carried by, for example, aphids (Berlandier, 1996; Adhikari et al., 2012). The end of the 20th century was marked by drastic onsets of Fusarium and anthracnose in all countries producing narrow-leaved lupine. The idea emerged to develop ‘bitter/ sweet’ cultivars, combining the bitterness of green biomass as a means of defense against pests and low alkaloid content in seeds to make them usable as feed or food (Wink, 1990; Philippi et al., 2015). Such idea could not be implemented without the knowledge of the entire multistep way of alkaloid biosynthesis which starts within the chloroplasts of young lupine leaves (Wink, Hartmann, 1982; Bunsupa et al., 2012), from where they are transported through the phloem into the generative organs (Lee et al., 2007). Recent research on the expression of genes responsible for alkaloid biosynthesis has shown that such biosynthesis is completely or nearly absent in the seeds, which confirms the transport of alkaloids from other tissues (Otterbach et al., 2019).

Biosynthesis of quinolizidine alkaloids has been studied to a much lesser extent than that of some economically important alkaloids in other plants that represent the model species for better understanding of this process (Nicotiana spp., Papaver somniferum, etc.). That is why the attempts have been made to gain an insight into the ways of lupine alkaloid synthesis and transport, using the knowledge of the synthesis of other alkaloids and the searches for homologous genes (see the reviews by Bunsupa et al., 2012; Kamel et al., 2016; Frick et al., 2017; Romanchuk, Anokhina, 2018).

Along with approaching these breeding tasks, which prospectively can be solved through the use of new reverse breeding or genome editing technologies, they are trying to modernize the centuries-old experience in the removal of bitterness from lupine seeds and green biomass. They develop the techniques of alkaloid extraction from large amounts of raw plant produce yielded by bitter cultivars, thus making it fit for animal feed. In 2013, for example, Russian researchers developed and patented the cost-effective biotechnology of profound lupine grain processing in a milk serum medium. Such line may be installed into the technological process of any compound feed producing factory (Korol, Lakhmotkina, 2016b). Thermal seed treatment with alkaline solutions could reduce alkaloid concentrations in seeds to 0.003 % (Jiménez-Martínez et al., 2001). In Portugal, at the enterprises that extract alkaloids from large bulks of lupine and consume lots of water, a trial was conducted to test the technology of discharged water detoxification by nanofiltration and binding of 99 % of lupanine contained in it, so that the latter could be used as raw material for the pharmaceutical industry (Barbeitos, 2016).

Thus, at present there are two ways to obtain alkaloidfree raw produce of narrow-leaved lupine for food and feed purposes: lengthy and intricate development of low-alkaloid cultivars by conventional breeding techniques, and novel technological lines of alkaloid removal/ extraction. The solution for lupine breeding is seen in the genome-based biotechnologies, as their certain prospects for narrow-leaved lupine are quite obvious.

## Genetic and genomic resources of narrow-leaved lupine

Intensification of breeding practice requires rich and diverse source material. A number of the world’s genebanks maintain the global diversity of L. angustifolius. The largest collections are in Australia, Poland, Portugal, and the Russian Federation. The Australian collection includes mostly wild lupine forms, recombinant inbred lines, mutant populations, and interspecies hybrids. These resources are used to study the genetic and molecular control over the key traits, and this work is expected to be reinforced by the ongoing research into L. angustifolius genome sequencing. The main objective of Australian researchers is to expand the genetic base of the species, including the involvement of wild lupine forms. Marker-based introgression of the desired traits is proposed (Berger et al., 2013). The marker-assisted selection has already become an integral element of Australian breeding programs and accelerated the development of new cultivars (Rychel et al., 2015).

Genetic maps have been produced for narrow-leaved lupine (Yang et al., 2013; Kamphuis et al., 2015) as well as vast libraries of genomic insertions (Gao et al., 2011). Genes responsible for the expression of economically useful traits, alkaloid content included, have been discovered and mapped (Boersma et al., 2005; Bunsupa et al., 2011).

The only one recessive iuc gene, out of the five known ones that determine the alkaloid content in narrow-leaved lupine, is used in breeding programs. The gene’s molecular functions have not yet been identified. Markers have been found for the locus iuc, and the denser cartographic resources and genome annotation have narrowed the region of the iucundus candidate gene (Li et al., 2011; Hane et al., 2016). The NGS (next generation sequence) technology is applied for more rapid development of markers for breeding (Yang et al., 2015).

The paths of alkaloid synthesis are partially known. However, their genetic base still remains poorly studied. Transcriptome sequencing (RNA-seq) and analysis of differentially expressed genes in a sample containing bitter and sweet narrow-leaved lupine accessions helped to detect 13 genes presumably involved in the synthesis of quinolizidine alkaloids (Kamel et al., 2016). The identified alkaloid biosynthesis genes were mapped, but only one transcriptomic factor from the RAP2 family of factors regulating secondary metabolism was closely linked with the iuc gene (Kroc et al., 2019). Investigating the mapping populations with the technique of massive analysis for cDNA ends (MACE) confirmed the idea that the ETHYLENE-RESPONSIVE TRANSCRIPTION RAC2-7 gene factor could control the low-alkaloid phenotype in narrow-leaved lupine (Plewiński et al., 2019).

## Conclusion

The gene pool of narrow-leaved lupine should become the target of more intense research on the phenotypic and genotypic levels, so that its diversity would be more obvious and available to plant breeders. This will help to optimize the development of cultivars with the desired properties. With this in view, it seems rational to make green manure cultivars high-alkaloid, but those intended for food and feed must not lose their adaptability-related traits, including pathogen resistance, at the expense of eliminated alkaloids. In this regard, a productive idea is to produce ‘bitter/sweet’ cultivars, combining high alkaloid content in their green biomass with a low alkaloid level in seeds. Its implementation depends on the knowledge of alkaloid biosynthesis and transport pathways in a plant and the possibility of their regulation, which seems a task for the nearest future, considering the currently available genomic resources. However, at the present moment it is not expedient to discard routine technologies of raw plant produce processing, using alkaloid extraction techniques with concurrent isolation of valuable ingredients for the pharmaceutical industry. Genetic resources–phenotyping-metabolomics–conventional breeding practice; genomic resources–marker-assisted and genomic selection/ genome editing; and cost-effective technologies of alkaloid extraction from the raw produce of bitter cultivars – these approaches are, in our opinion, the best to improve the economic potential of this valuable pulse crop and make use of it in the present-day situation and in future.

## Conflict of interest

The authors declare no conflict of interest.
